# Recent innovations of ultrasound green technology in herbal phytochemistry: A review

**DOI:** 10.1016/j.ultsonch.2021.105538

**Published:** 2021-03-25

**Authors:** Mostafa Gouda, Alaa El-Din Bekhit, Yu Tang, Yifeng Huang, Lingxia Huang, Yong He, Xiaoli Li

**Affiliations:** a College of Biosystems Engineering and Food Science, Zhejiang University, 866 Yuhangtang Road, Hangzhou 310058, China; b Department of Nutrition & Food Science, National Research Centre, Dokki, Giza, Egypt; c Department of Food Science, Otago University, New Zealand; d College of Automation, Guangdong Polytechnic Normal University, Guangzhou 510665, China; e College of Civil Engineering and Architecture, East China Jiaotong University, Nanchang 330013, China; f College of Animal Sciences, Zhejiang University, Hangzhou 310058, China

**Keywords:** Ultrasound phytochemistry, Sonochemistry, Herbal nanotechnology, Acoustic-based biosensors, Green technology

## Abstract

Ultrasound (US) has become one of the most important techniques in green chemistry and emerging technologies. Many research investigations documented the usefulness of US in a wide range of applications in food science, nanotechnology, and complementary medicine, where effective extraction of natural products is important. However, as with all novel technologies, US has advantages and limitations that require clarification for full adaptation at an industrial scale. The present review discusses recent applications of US in herbal phytochemistry with the emphasis on US effects on chemical structures of bioactive compounds extracted from herbs and their bioactivities. The impact of different US processing conditions such as frequency, intensity, duration, temperature, and pressure on the effectiveness of the extraction process and the properties of the extracted materials are also discussed. Different frequencies and intensities of US have demonstrated its potential applications in modifying, determining, and predicting the physicochemical properties of herbs and their extracts. US has important applications in nanotechnology where it supports the fabrication of inexpensive and eco-friendly herbal nanostructures, as well as acoustic-based biosensors for chemical imaging of the herbal tissues. The application of US enhances the rates of chemical processes such as hydrolysis of herbal fibers, which reduces the time and energy consumed without affecting the quality of the final products. Overall, the use of US in herbal science has great potential to create novel chemical constructions and to be used as an innovative diagnostic system in various biomedical, food, and analytical applications.

## Highlights

• Ultrasound enhances the phenolic compounds in extracted herbal oils by 44%.
• US (25–50 kHz) increases the yields of polyphenols, carotenoids, and flavonoids.
• Radicals generated by US have positive and negative effects on enzymes.
• US improves the efficiency of herbal extracts in Nanotechnology applications.
• Acoustic-based biosensors could be used for chemical imaging of herbal tissues.

## 1 Introduction

Integrating physical and chemical technologies for the characterization and modification of natural plants, like herbs and spices, has been used for several decades to improve their potency and quality [1], [2]. Herbs have a long history of medicinal uses that have been documented by their traditional uses and recent scientific evidence, in particular in China. Chinese herbs have been traditionally used for the treatment of different disorders, such as respiratory and heart diseases and mental disorders. During the SARS epidemics, traditional Chinese herbal medicine treatments were reported to have positive therapeutic effects on SARS [3], [4]. Scientists have been exploring the scientific basis and mechanism of action of herbal medicine with great interest in their chemical constituents [5]. Special attention has been paid to investigate the different methods for the preparation and processing of herbs to maximize the efficacy of their bioactivities and to understand the changes in their chemical compositions [6].

Ultrasound treatment is an acoustic technology that can be used for non-invasive detection and/or modification of herbal bioactive compounds. As a physical treatment, it can modify the chemical and physical properties of biological systems at varying levels depending on the processing conditions (e.g. frequency, intensity, and duration) and the herb's structure and composition [7]. The technology is a green technology that offers opportunities to create new functional products or extract more powerful functional bioactive compounds from herbs. Also, it is a low energy and maintenance cost technology that has several economic benefits [8]. However, US is not a largescale standardized technology that could be adapted at a commercial scale for modification of herbs and other foods. A better understanding of the complex relationships among the processing conditions (frequency, power, and processing duration) and herbs structures and chemical compounds can support future applications in the herbal industry [9]. On the other hand, phytochemical changes can occur by free radicals that are generated by cavitation caused by the US shock wave energy. These changes in phytochemicals are considered a huge challenge for US technology. Collectively, recent research suggested that US can be considered as a viable technology in quality assurance and food safety applications [10], which adds greater benefit for the optimization of the latest extraction technologies [2].

This review aims to provide a comprehensive review of mechanisms of action of ultrasound and their effects on the quality of herbal products. A special focus has been placed on the relationships among the tested materials, mechanisms of action, techniques, and processing conditions.

## 2 Herbs and spices definition

Herbs and spices are botanical raw materials that have active components that could be used in pharmaceutics, cosmetics, food additives, and health supplements [11]. Herbs are used by complementary medicine therapists to treat various diseases. Many plant-based poly or mono herbal formulas are used for various non-communicable diseases like cardiovascular diseases and cancers [12]. The words “herbs” and “spices” have many definitions but the most common are those which consider herbs to be obtained from the green parts of a plant, for example stems and leaves such as tea, mints, and thymus. On the other hand, spices are produced from other structures such as seeds, flowers, fruits, barks, or roots, for example garlic, turmeric, cinnamon, and black pepper [13], [14], [15]. These plant materials have high contents of phytochemicals; including phenolic, carotenoid, flavonoid, and volatile components that exhibit antimicrobial, antioxidant, and other biological activities [[16], [17], [18]]. The long historical use of herbs and spices document their safe use, thus they are generally recognized as safe (GRAS) materials. This is a great advantage to use herbs and spices as natural alternatives to chemical additives [19].

## 3 Ultrasound technology

Ultrasound is defined as any mechanical sound waves at frequencies >20 kHz, which is beyond the human hearing threshold. Compression and expansion waves generated by US lead to positive and negative pressures that can create cavities in the treated materials [20]. The formed cavities release high energy and generate high-pressure and localized extremely high temperatures [21]. The produced shock wave energy hydrolyzes water into free radicals (H· and ·OH) and forms hydrogen peroxide (H_2_O_2_) as a byproduct. The main parameters that control the effectiveness of the treatment are the US velocity, attenuation, and acoustic impedance [22].

### 3.1 Ultrasound generation and treatments

The US waves are formed from the conversion of mechanical oscillation of a high-frequency electrical field, which has an elastic effect (stretching and compressing) on the deformation of ferroelectric materials [23]. For materials' physicochemical measurement, the US transducer emits pulses against one side of the material being analyzed and these waves are transmitted into the material. The time that the pulses take to pass through the material and return to a detector is measured and the echo velocity in the material is calculated [24]. This phenomenon is affected by several factors, such as the chemical composition and the structural configuration of the material.

Ultrasound can be categorized according to the used frequency (kHz) and the generated energy intensity power (W). Also, it could be categorized based on sound intensity (W/m^2^) or sound energy density (Ws/m^3^) [23]. According to the used frequency, it can be categorized into high and low frequencies. High-frequency and intensity US are those using frequency higher than 100 kHz, and intensity from 10 to 1000 W/cm^2^, whereas the low-frequency and intensity US uses frequency lower than 100 kHz, and intensity lower than 3 W/cm^2^ [[25], [26]].

**Fig. 1:**
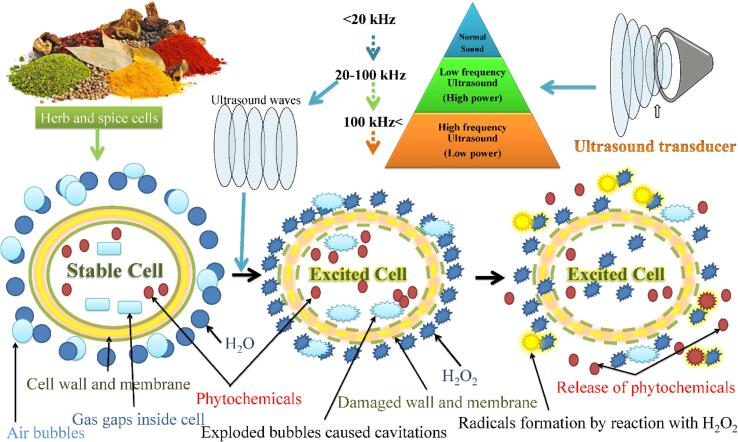
Principle effects of low-frequency and high-intensity ultrasound on herbs and spices cells.

Different combinations of frequency and intensity power are used for different US applications. High-frequency and intensity ultrasound combination is generally used for non-food applications such as soft tissue surgery, diagnostic imaging, or drug delivery [27]. Similarly, high-frequency and low-intensity US is used in the simulation of tissue regeneration [26]. Low-frequency US at low or high-intensity combinations is generally used in food applications. The intensity plays a key role in forming stable cavities and imploding them (Fig. 1). The low-intensity US is used as a non-destructive technique for providing information about the materials' physicochemical properties, like structure, composition, and physical state. On the other hand, high-intensity US is used to change the chemical or physical properties of materials such as generating emulsions, promote certain chemical reactions, or change the functionality of proteins and carbohydrates [24]. Low frequency and high-intensity US has been used in research to study the composition of vegetable, fruit, milk, meats, gels geopolymers, molecular interactions, and protein structures [22]. The high-intensity US has been used to reduce microorganisms and to facilitate meat tenderization.

There are three commonly used US transducers: liquid-driven, magnetostrictive, and piezoelectric transducers. Modern instruments based on piezoceramic US transducers are considered very important because of their high acoustic properties like producing high sound pressure amplitudes by simultaneous small power consumption [28].

### 3.2 Advantages and disadvantages of different US treatments

Different US processing conditions can exert several benefits in the field of herbal science. For example, high-US penetrating power allows the detection of flaws deep in the plant parts. US increases the efficacy of the herbal extraction methods at lower temperatures and improves the rates of heat and mass transfer, cell disruption, and the penetration of solvents to the herbal tissues [[29], [30]]. Also, it speeds up the filtration process, increasing the life of the filter, results in a faster drying process, and thawing operations [31]. Bellumori, Innocenti, Binello, Boffa, Mulinacci and Cravotto [32] demonstrated that high-intensity US (titanium horn, 19.5 kHz,140 W) is a rapid, efficient, and selective technique for rosemary (*Rosmarinus officinalis*) leaf extraction and provide extracts with high bioactive compounds such as rosmarinic and carnosic acids. The application of US in herbal extraction showed a significant reduction in processing time compared to the conventional methods and the obtained extracts had slightly higher antimicrobial activity against some pathogenic species [33]. O’Donnell, Tiwari, Bourke and Cullen [34] stated that the low-intensity US has a high ability to monitor the properties during processing as well as being non-destructive, rapid, and precise for characterizing food and plant complexes.

On the other hand, high-US intensities (>400 W) generate heat and increase the treatment medium temperature, which may cause adverse physical and chemical effects on some herbs' phytochemicals. Thus, the intensity and energy of US should be optimized before application on different plant tissues [31]. Further, free radicals generated due to cavitation may result in several negative changes such as lipid oxidation accompanied by off-odor compounds, protein denaturation/ oxidation, and reduction in total phenolic contents [35].

## 4 Application of US in herbal science

The application of US in herbal science is increasing due to its significant effect on the bioactive compounds in the herbs. Table 1 presents recent applications and the impact of different ultrasonic frequencies, intensities, and duration as well as their major effects on herbs and spices.

**Table 1:** Summarization of recent herbal plant treatments by using ultrasound in different application fields.

Samples name	Scientific Name	Process	US method	Compounds of interest	Freq. (kHz)	Int. (W)	Time (Min)	T. (°C)	Ultrasound effects	Ref.
Alfalfa	*Medicago sativa*	Ultrasonic bath	Thermosonication, structural study	Saponins	–	50–150	1–3 h	50–80	Ultrasound treated samples yield rate increased almost two times compared to the heat-reflux method. Also, it has greater efficiency for the saponins yield and bioaccessibility.	[36]
Arrowhead	*Sagittaria sagittifolia*	Sonication chamber with ultrasonic generator	Thermosonication treatment	Bioactive proteins	28–40	60 W/L	10–60	30–50	The results showed that ultrasound treatment had a considerable impact on the protein structure and it could increase the protein susceptibility digestive enzymes like pepsin and trypsin. In which, us is providing a powerful endorsement for increasing the protein proteolysis.	[37]
Clove Tarragon	*Syzygium aromaticum* *Artemisia dracunculus*	Ultrasonic horn and probe sonotrode system	Thermosonication, extraction and application in nanofiber technology	Essential oils	53	500	20–40	32–52	Thermosonication showed the most important influence on the extraction yield. Also, for tarragon, the antioxidant activity is increased at 250 W however it decreased with increasing the power until 500 W because of the destructive effect of ultrasonic treatment at high intensity.	[[38], [39], [40]]
Chinese ginseng	*Panax Notoginseng*	Ultrasound bath	Sonication extraction	Saponins	–	–	20	–	Compared with traditional extraction method, ultrasound has enhanced the four saponins yields. With potential advantages involving shorter extraction time, it decreased the consumed solvent.	[[41], [42]]
Chinese liquorice Gancao Kenfa	*Glycyrrhiza uralensis* *Turpiniae folium* *Hibiscus cannabinus*	Ultrasonic probe sonotrode system	Polysaccharide thermosonication extraction and modification	Polysaccharide, Cellulose	25	0–600	10–60	25–100	For glycyrrhizauralensis as thermal stable polysaccharide, the optimal extraction parameters of glycyrrhiza polysaccharide is 600 W for 60 min at 70 °C. And for *turpiniae folium* is 200 W for 30 min at 30 °C	[[43], [44], [45]]
Citrus limon leaves	*Citrus aurantium*	Ultrasonic probe sonotrode system	Sonication for nanocubes and nanosphere nanocomposite	Phytochemical mixture	20	250	29		In this work, ultrasound synthesized an efficient photocatalyst nano-cubes and nanospheres by using C. limon LE and Ag:CdO. In which, C. limon phytocomponents played an effective role as reducing and stabilizing agent.	[46]
Coriander leaves	*Coriandrum sativum*	Cylindrical jacket with in-built piezoelectric US transducer rode	Synthesis of iron oxide nanoparticle	Phytochemical mixture	33	–	–	20–60	US assisted green synthesis of iron oxide nanoparticles using coriander extract as a reducing agent. In which, it showed higher antioxidant and antimicrobial activity compared to the conventional method due to the combined effect of US and the plant molecules attached with iron oxide nanoparticles.	[47]
Cumin seeds	*Syzygium cumini*	Ultrasound bath	Thermosonication, extraction	Catechin, Gallic acid	22 , 40	44–215	0–20	25–65	Catechin and gallic acid extracted yields were increased by 3.7 and 2.1 times with increasing US power from 44 to 125 W. However, the yields were not significantly increased over 125w power and 35°C.	[48]
Erodiumherb Lettuce herb	*Erodium laucophyllum* *Lactuca sativa*	Ultrasonic probe sonotrode system	Sonication extraction for antimicrobial evaluation	Phytochemical mixtures	26	200, 400	5,10	40,45	Ultrasound showed the highest level of desirable phenolic compounds. In which, exhibit the highest level of antimicrobial and antiviral activities especially against hepatitis a and murine norovirus. Also, it increased the decontamination of lettuce herb.	[[49], [50]]
Garlic peels	*Allium sativum*	Ultrasonic disperser	Thermosonication treatment	Polysaccharide	40	500	0–30	65	Ultrasound increased C═C bond and c/o ratio, which is beneficial to improve the electrochemical function of materials	[51]
Green tea	*Camellia sinensis*	Piezoelectric ceramic ultrasound bath, horn, probe sonotrode	Sono-solid-phase microextraction , hydrolysis, and physicochemical and functional properties studies	Volatile compounds	20 kHz, 1.7 MHz	100	15–120	20–70	Ultrasound increased the polyphenols, flavonoids, and volatiles concentrations of medium-to-low volatility fractions which may function as an assisted tool to volatiles extract from tea herb. Also, hydrolysis efficiency for tea reducing sugars production is increased by US.	[[9], [52], [53], [54], [55]]
Hibiscus	*Hibiscus sabdariffa* *Hibiscus* tiliaceus	Ultrasonic cleaner bath	Sonication nanostructures	Chlorophyll and other phytochemical mixtures	500 Hz, 40 kHz	130	30–60	–	Ultrasound can be used for fabricating of inexpensive, simple, eco-friendly hibiscus with ZnO and other nanostructures to extend their utility in different areas of nanotechnology.	[[21], [56]]
Mulberry leaves	*Morus alba L.*	Ultrasonic probe sonotrode system	Ultrasonic drying process	Phytochemical mixture	20	130	5–15	20	US pretreatment enhances mulberry drying kinetics and reduces total energy consumption without affecting product quality.	[57]
Parsley leaves	*Petroselinum crispum*	Ultrasound bath	Ultrasonic drying preprocess	Phytochemical mixture	21	100–300	20	30	US pretreatment significantly reduced the drying time and consumed energy up to 29.8% and 33.6%. Also, it increased parsley chlorophyll a and b resistance to the drying process.	[58]
Peppermint leaves	*Mentha piperita*	Drying chamber with piezoelectric US transducer (1500 W)	Ultrasonic drying process	Phytochemical mixture	20	90–360	40–400	40–70	US improved the convective drying of peppermint. In which, The results showed that it decreased energy consumption and increased energy efficiency up to 3.69%.	[59]
Rosemary	*Rosmarinus officinalis*	Ultrasonic titanium horn	Sonication extraction	Rosmarinic Carnosic	19.5, 35	140, 320	15	25	Increased efficiency and decreased processing time	[[60], [61]]
Scarlet Sage	*Salvia coccinea*	Ultrasonic homogenizer bath	Sonication extraction	Polyphenols	50	–	15–450	–	Ultrasonic assistant extraction shortened the processing time and provided lower solvent consumption	[62]
Thyme	*Thymus serpyllum*	Ultrasonic horn	Ultrasonic drying and extraction processed	Polyphenols	20	750 W, 6.2–18.5 kWm^−3^	–	25–80	Enhanced product quality and shorted time and decreased processing cost of thyme herb. Also, US increased total polyphenols and flavonoids yields and extraction efficiency from *Serpylliherba* or wild thyme.	[[63], [64], [65]]

### 4.1 Ultrasound applications targeting the chemical composition of herbs and spices

**Fig. 2:**
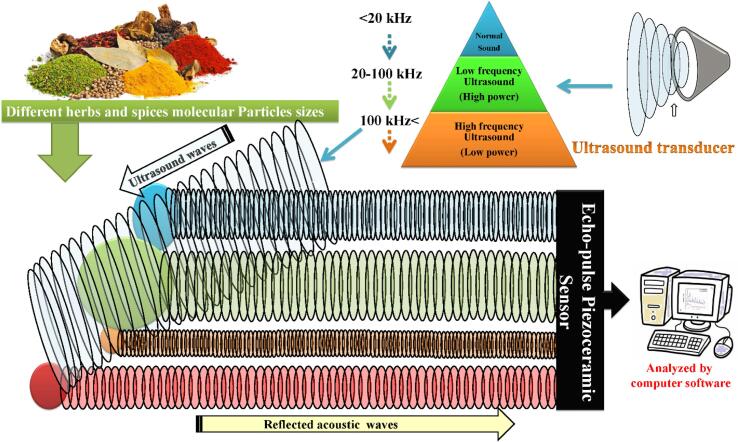
Principle effects of high-frequency and low-intensity ultrasound in measuring particle size of molecules.

Ultrasound is useful for food composition measurement because it is non-destructive, rapid, and could be adapted for optically opaque systems. For instance, low intensity (<1 W/cm^2^) or high-frequency (>100 kHz) US were used to obtain detailed information about the structure, dimensions, and composition of the plant products during the storage process [66]. In which, differences in the chemical composition produce different responses to US properties (velocity, attenuation, frequency, and power). In measuring particle size, US uses the same principles of light scattering in emulsions or suspensions. In which, US velocity and attenuation coefficient depends on the size and concentration of particles (Fig. 2).

Despite the beneficial effects of the US, it has been observed that US promotes several oxidation reactions and enzyme inhibitions of many food enzymes, including peroxidases, and glucosidases [67]. This is probably because of the intense pressures, temperatures, and shear forces generated by the ultrasonic waves that denature proteins [10]. Furthermore, the extreme agitation created by microstreaming could change Van der Waals interactions and hydrogen bonds in the polypeptides resulting in protein denaturation such as that observed in *Dolichos lablab* [68]. Free radical-mediated deactivation mechanisms and cleavage of the functional groups from enzymes could occur during US treatment and is the likely reason for the modifications in proteins and enzymes. For example, the inactivation of peroxidase as a result of haem group dissociation and the loss of iron was facilitated by hydroxyl radicals from US cavitation [69].

### 4.2 Recent mechanistic insights of US process on herbal chemicals composition

**Fig. 3:**
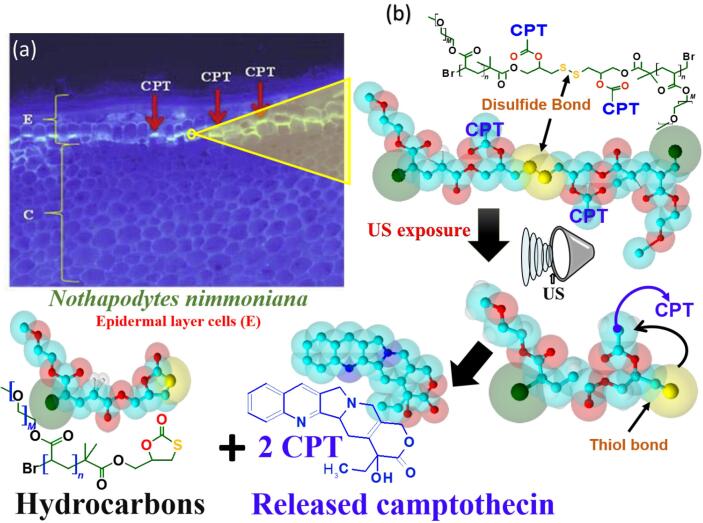
US mechanochemistry releases camptothecin (CPT) herbal monoterpene. (a) Fluorescent micrograph of *Nothapodytes nimmoniana* stem sample showing autofluorescence of CPT in the epidermal layer. (b) The US mechanochemical cleavage of polymers disulfide bond that releases CPT from its β-carbonate linker presented in 2D and 3D structures (License Number: 5022331159441) [[71], [72]].

The effects of US depend on the changes in the chemical structure of the material being treated. Heterogeneous reactions that involve unfragmentable substrates inside the US bubbles have been shown to occur by the mechanical effects of the US and lead to increasing chemical reactivity [70], a relationship that is known as mechanochemistry. Huo, Zhao, Shi, Zou, Yang, Warszawik, Loznik, Gostl and Herrmann [71] reported that US mechanochemistry can be exploited to control transformations at the molecular level by rearranging or cleaving bonds at predetermined breaking sites. A recent study investigated the impact of US on camptothecin (CPT) (plant alkaloid monoterpene produced by *Camptotheca acuminata* herb) and the authors found that US transformed the disulfide bonds to thiol bonds of the molecules. As a result, US modified the molecules from covalently attached linear polymer chains in the β-position to a disulfide moiety (Fig. 3b) [71].

The position of the disulfide motif in the center of the macromolecular framework (e.g. Hydrocarbon) enabled the efficient mechanochemical scission and the release of this monoterpene molecule [69]. A US mechanophore breakage (mechanochemically breakage of the polymer reactive units like cyclic rings) can occur in extracts solutions via the shear stress caused by the collapse of US-induced cavitation bubbles [[73], [74]]. These modifications in the structure of extracted compounds caused US can facilitate better bioavailability of the herbal bioactive molecules [75]. Patil and Akamanchi [72] used US (20 kHz, 150 W, 30 °C) for CPT extraction from the stem part of *Nothapodytes nimmoniana* herb. The application of the US increased the camptothecin yield (1.7-fold) and decreased the extraction time from 6 h to 18 min. The authors suggested that the mechanochemical effects of the US disrupted both the outer and interior parts of stem cell wall structure (Fig. 3a). The bubble collapse creates high-speed solvent jets that disassociated the disulfide and covalent bonds on the surface and interior parts of the cells which release the CPT [72] (Fig. 3b).

### 4.3 Ultrasound effects on herbal phenolics and polyphenols

Ultrasound has a significant influence on plant phytochemicals, especially polyphenols [30]. For example, using US for extraction of gingerols from ginger powder at 50 °C enhanced the extraction of gingerol yield. However, a degradation of the active compound occurred at higher temperatures [76]. Boulatov [75] reported that the mechanochemical effects of the US results in overstretching of macromolecules polymers (like carbohydrate and protein chains) that lead to their fragmentation. This stretching process helps in releasing small molecules that are bound in the polymer chain. A comparison study of US-assist and conventional extraction methods on herbal tea bioactive compounds showed that total phenols and α-tocopherol were increased by 44% and 20%, respectively. The breakdown of cells cytoarchitecture has been found to release these bioactive compounds [77]. According to Ranalli, Malfatti, Lucera, Contento and Sotiriou [78], the increase in bioactive components in oil extracted by US could be due to the breakage of cross-links between these compounds and other macromolecules like polysaccharides and protein. Also, Rashed, Tong, Abdelhai, Gasmalla, Ndayishimiye, Chen and Ren [79] reported that higher total phenolic content was extracted from *Lavandula pubescent* herb using US compared to a maceration extraction. For most herbs, the US acoustic cavitation can facilitate the flow of solvent into the plant cells, and enhance desorption of bioactives from the matrix of solid samples. Thus, US enhances the efficiency of phenolic extraction. In another study that investigated *Elsholtzia ciliata* herb, Pudziuvelyte, Jakštas, Ivanauskas, Laukevičienė, Ibe, Kursvietiene and Bernatoniene [80] reported that US significantly increased the extracted apigenin phenolic yield (855.54 μg/g) compared to the maceration method (141.06 μg/g). The authors reported that US-assisted extraction for 11 min increased the mass fraction of total phenols by 20% compared to water bath shaker for 30 min with the same solvent. Also, US treatment increased chlorogenic acid content up to 2174.70 μg/g after 30 min compared to the percolation extraction method that resulted in 683.40 μg/g [80].

### 4.4 Ultrasound effects on herbal carotenoids

Using of US has its significant effect on the carotenoids content in oil extracted from herbs. For example, Hu, Li, Qin, Zhang, Liu, Zhang, Liu, Jia, Yin, Han, Zhu, Luo and Liu [77] reported that application of US (25 kHz, 550 W, 70 °C for 38 min) in the extraction of tea (*Camellia sinensis*) oil increased β-carotene by 38% compared to conventional extraction methods. The authors suggested the increase was due to the breakage of cross-links between these compounds and other macromolecules like polysaccharides and proteins, which release the carotene in the extraction medium. However, high-intensity US application (25 kHz and 600 W at 4 °C for 6 min) significantly degraded (all-E)-astaxanthin carotenoid [81]. Additionally, after optimization of US parameters to extract antioxidants from thyme (Thymus vulgaris) and rosemary (Rosmarinus officinalis), Munekata, Alcantara, Zugcic, Abdelkebir, Collado, Garcia-Perez, Jambrak, Gavahian, Barba and Lorenzo [82] reported that US-assisted extraction at 400 W and 40 °C for 10 min increased the extraction yields of carotenoids compared to conventional extraction (heating under magnetic stirrer method). Also, the authors mentioned that US improved the aqueous extraction of antimicrobial compounds from thyme.

### 4.5 Ultrasound effect on herbal flavonoids

While the formation of free-radicals during US processing is considered a main disadvantage of the technology as it affects the bioactivity of components such as phenols [83], chemical modification such as increasing the extent of hydroxylation of flavonoids [84] can enhance the antioxidant activity or at least counteract some of the negative effects of free radicals. This led to the fact that optimum US processing conditions where maximum yield and bioactivity could be optimized for various materials. In which, US inhibits the hydrolyzing enzymes (such as α-amylase and α-glucosidase) which affects the total flavonoids yields and their antioxidant functionality [67]. For instance, the extraction efficiency of dihydromyricetin yield, which is the main flavonoid in Chinese vine herbal tea (Ampelopsis grossedentata), was increased up to 40% (3% yield) with the increase in US power and time until a certain extent (5.5 min at 240 W), after which, the yield was decreased due to hydrolysis of the compound [85]. Similarly, total flavonoids extracted from *Syzygium cumini* seeds using US (22–24 kHz and 44–215 W at 35 °C for 12 min) were increased with the increase in processing time [48]. Maximum total flavonoid content was obtained after 12 min of processing at US power of 125 W at 35 °C for 12 min. The yields of gallic acid and catechin exhibited similar trends with 54.5 and 2.2 mg/g after 12 min [48]. Collectively, the above information suggests a scope for optimization where maximum bioactives could be obtained before negative effects are caused by heating or free radicals.

**Fig. 4:**
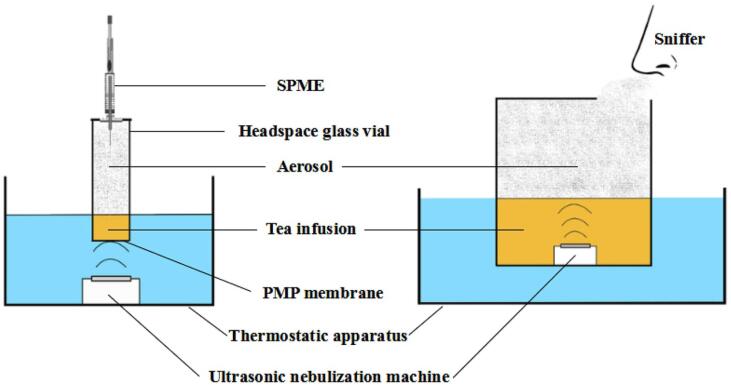
Schematic of the US-nebulization extraction device used to extract volatile compounds (left) and to evaluate aroma (right). SPME, Solid Phase Microextraction; PMP: Polymethylpentene membrane [9] (License Number: 4958661463662).

Optimum US extraction conditions for total flavonoids from sour jujube seeds (*Caenorhabditis elegans*) were obtained at 404 W and 60 °C for 60.03 min. The use of US increased the flavonoids' yield by 17.11% compared to heat reflux extraction. Moreover, the US extracted flavonoids showed higher antioxidant capacity against DPPH, superoxide, and hydroxyl radicals from the significant differences in their chemical construction due to using US-assisted extraction (UAE) or heat reflux extraction (HRE) (Fig. 4) [86].

### 4.6 Ultrasound effect on essential oils and other volatiles

The effects of ultrasound on plant essential oils have been recently studied [87]. Essential oils are unstable to heat, thus, the use of US at low intensities could prevent the degradation of these thermally sensitive compounds. Furthermore, excellent diffusion rates were afforded by the use of US, which can facilitate successful extraction with minimum solvent use and subsequently lower solvent residues in the extracted compounds. The composition of the extracted essential oil could be modified and more selective extraction of desired compounds could be obtained [30]. For example, by using 20 kHz and 90 °C for 70 min of US, red pepper seeds' alcohol and aldehyde contents were decreased, while other volatile components, such as pyrazine derivatives, esters, and olefin components were increased [88]. These changes in the composition of the extract could eventually have subsequent effects on the efficacy of the extracts and potentially on their sensory attributes.

For Lu'an Guapian tea herb (*Camellia Sinensis*), Meng and Zhengquan [9] established a method for extracting volatiles by US nebulization extraction (UNE) combined with solid-phase microextraction method (SPME) (Fig. 4). In that study, the authors reported that the extracted total volatiles percent was increased significantly (*p* < 0.05) to 42.23% by using 1.7 MHz and 50 °C for 20 min of US. A significant increase in some aldehydic volatiles such as pentanal, heptanal, octanal, and dodecanal was also found in the obtained extracts compared to controls [9].

It is worth mentioning that the extraction conditions play an important role in determining the composition of the volatiles. For example, while no significant differences were observed in alcoholic volatiles such as hexanol at 20 °C, increasing the extraction temperature to 50 °C resulted in a significant increase in alcoholic volatiles, such as α-terpineol. Therefore, US can increase the volatile extraction efficiency of medium-to-low volatiles compounds in herbs [9]. The combined use of US and heat treatment using a thermosonication method improved the extracted essential oil efficiency and extracted yield from clove (53 kHz and 52 °C) [38]. Also, time savings have been reported for the US processing where Gavahian, Farhoosh, Javidnia, Shahidi, Golmakani and Farahnaky [89] reported of reducing essential oil extraction time by 17% for peppermint herb.

#### 4.6.1 Ultrasound effect on terpenes

It has been shown that thermosonication can significantly increase the percentage of some extracted terpenes from herbal sources [9]. For example, a significant increase in β-ocimene, and D-limonene contents of tea (*Camellia Sinensis*) were reported as a result of using 50 °C of US (1.7 MHz, 20 min) compared to 20 °C. On the other hand, alcoholic terpenes such as α-terpineol were not affected by US treatments [9]. As mentioned earlier, CPT extraction from the *Camptotheca acuminata* herb by US transformed the disulfide bonds to thiol bonds of these molecules, which resulted in the disulfide motif to be in the center of the macromolecular framework (e.g. Hydrocarbon) and enabled efficient mechanochemical scission and the release of this monoterpene molecule [69]. High-intensity US using a titanium horn at frequency 19.5 kHz and intensity 140 W increased the extraction efficiency of carnosic (13%) and rosmarinic (6.8% of dry extract) acids from rosemary leaves [[60], [61]]. Similarly, it has been found that US increased the efficiency of terpenes extraction from oregano herb, especially carvacrol (a monoterpene), ursolic acid (a triterpenoid), and oleanolic (a triterpenoid saponin) compared to the maceration conventional method [90].

### 4.7 Ultrasound effect on herbal proteins

Ultrasound treatment affects the protein structure in herbs due to its modifying effect on some secondary bonds of β-sheets and β-turns, which affects the protein's hydrophilic groups and the hydrophobic core [91]. A study on arrowhead (*Sagittaria sagittifolia*) herbal protein showed that US treatment (28–40 kHz, 30–50 °C) caused unfolding of the protein structure, decreased α-helix and β-turn contents, and increased β-sheet and random coil contents. In which, the content of free sulfhydryl (SH) increased by 38.87% at 40 kHz and 40 °C over the control samples [59]. Also, it can enhance the formation of peptides subunits which formed as a result of plant proteins enzymolysis [37]. The overstretching of folded macromolecule disrupts the non-covalent interactions responsible for its three-dimensional configuration and affects its ability to be involved in interactions, e,g, change in the active sites of enzymes [75]. For instance, Ayim, Ma, Alenyorege, Ali and Donkor [92] investigated the effect of US (20 kHz, 20–50 °C, 13 min) pretreatment on the enzymolysis of tea residue protein extracted with sodium hydroxide. The authors stated that Michaelis constant (substrate concentration required to half saturate the enzyme) in US pretreated enzymolysis was decreased by 32.7% over the traditional enzymolysis, which just uses sodium hydroxide. An increase in the protein susceptibility to the proteolysis enzymes like pepsin and trypsin was also suggested [37]. Hadidi, Khaksar, Pagan and Ibarz [93] reported that US enhanced alfalfa leaves (*Medicago sativa*) protein extraction based on the pH, temperature, and duration. A high-intensity US (20 kHz, 100 W, 30–50 °C for 120 min) changed alfalfa leaves protein physicochemical properties and caused an increase in the protein surface hydrophobicity through disruption of proteins' hydrogen bonds, which affects the proteins surface charge. That US treatment increased the proteins’ solubility due to forming hydrophilic soluble fractions. Also, US (20 kHz, 60 °C for 78.1 min) promoted the interaction between polycysteine and xylose sugar. This eventually increased the antioxidant properties and decreased the formation of sulfur-containing volatiles from the Maillard reaction that is commonly encountered in conventional extractions [94].

#### 4.7.1 Ultrasound effect on enzymes activity

In general, most studies reported that the high-intensity US can chemically and physically inactivate many kinds of enzymes [37]. Enzyme molecular weight is very important for the sensitivity of enzymes to the US. For example, polymeric enzymes are fragmented into monomeric subunits, and these monomeric enzymes could be subsequently fragmented further or form aggregates upon extended US treatment. Enzyme protein denaturation that inactivates enzymes is promoted by free radicals and shear forces caused by cavitation [95]. For instance, US inactivated pectin methylesterase, which hydrolysis pectin and results in a product with low stability [96]. A combination of US, pressure, and heat treatment (manothermosonication) showed the highest inactivation of this enzyme compared to sonication or thermosonication alone at the comparable intensity levels. The high inactivation rates by temperature and pressure could be due to their effects on pectin and other molecules that interact with the enzyme and their absence lead to reduced enzyme resistance against temperature and pressure effects [97].

For polyphenol oxidase which causes enzymatic browning of natural plants, Cheng, Soh, Liew and Teh [98] found that US (35 kHz, 20 °C for 15 min) treatment increased the enzyme activity, however, longer time (35 kHz, 20 °C for 30 min) have inactivated the enzyme (20% inactivation) due to its denaturation [99]. Moreover, peroxidases which are categorized as high thermal stable enzymes are associated with undesirable flavors and pigments loss was reported to be inactivated using appropriate processing conditions. In watercress herb (Nasturtium officinale), thermosonication (20 kHz, 40–92.5 °C) reduced its inactivation time from 70 to 5 s [100]. This change was related to changes in enzyme tertiary structure, which affected the enzyme prosthetic group [100].

Manosonication can be defined as a combination of US and high pressure, which can make additional effects with US. This method can significantly change the protein and carbohydrates' conformations and configuration at pressure (100–300 kPa) and low temperatures. Thermal processing with 20 kHz of US is reported to be effective against many enzymes, like dehydrogenase and catalase. Manothermosonication is a combination of US and both heat and pressure that lead to synergistic effects on the extraction of compounds, inactivation of enzymes, and microorganisms. Almost complete enzyme inactivation of heat tolerated enzymes can be at 70 °C, 300 kPa for 2 min [[101], [102]]. Moreover, it has been reported that manothermosonication has the potential to inactivate many enzymes that tolerate thermosonication. The inclusion of pressure enhances the action of US and heat in cleaving prosthetic groups of enzymes or denaturize protein subunits. For example, the splitting of the prosthetic heme group of peroxidase, which is the mechanism of heat inactivation, is reported for manothermosonication [103]. Also, manothermosonication was found to be more effective than heat treatment alone in the inactivation of heat-resistant protease and lipase secreted by *Pseudomonas fluorescens* [104].

### 4.8 Ultrasound effect on herbal lipids and phytosterols

Chemat, Grondin, Shum Cheong Sing and Smadja [105] studied the effect of various US frequencies (20 and 47 kHz) treatment during the processing of sunflower oil. The authors found significant negative changes in the oil composition (degradation of linoleic acid and sterols and increasing of aldehydic volatiles like hexanal and hept-2-enal), due to free-radicals oxidation during the treatment. On the other hand, the oil of red pepper (*Capsicum annuum*) seed was not affected by the US treatment (20 kHz, 30–90 °C for 70 min) [88]. The authors did not find changes in acid and saponification values after using US which indicates that US did not affect the oil molecular weight. However, the peroxide value was slightly increased due to slight oxidation of the oil [88]. Also, relatively high amounts of phytosterols (stigmasterol, sitosterol, and sitostanol) were observed after US treatment that was paralleled by a decrease in campesterol stability (38% loss) [88]. Hu, Li, Qin, Zhang, Liu, Zhang, Liu, Jia, Yin, Han, Zhu, Luo and Liu [77] showed that US (25 kHz, 550 W, 70 °C for 38 min) treatment for oil extraction from tea (*Camelia sinensis*) increased total phytosterols, β-sitosterol, stigmasterol, campesterol and other phytosterols by 20, 25, 16, 37 and 26%, respectively. Furthermore, Panadare, Gondaliya and Rathod [106] found that US pre-treatment (150 s at 30 W) increased oil yield by 11% from *Annona squamosal* seeds compared to conventional methods. The oil characteristics from US and conventional methods were not different in their acid value or free fatty acids. The Oleic / Linoleic ratio was 2.21 which is similar to the range reported in the literature [68].

### 4.9 Ultrasound effect on herbal carbohydrates

Herbal polysaccharides, as natural macromolecules, have been demonstrated to have significant bioactivities, such as anti-inflammatory, antimicrobial, and antioxidant activities [107]. The application of US can enhance the medicinal properties of polysaccharides but the extent of improvement is dependent on the treated herbs. For instance, Zhao, Xia, Lin, Xiong, Tang and Liao [44] showed that US has a significant effect on polysaccharides extracted from dried leaves of Chinese herb *Turpiniae Folium* and optimum extraction conditions for high yield were 200 W at 30 °C for 35 min.

US (45 kHz) at different power levels (40–100 W) were used extraction of polysaccharides from *Acantho panaxsenticosus* herb and their antioxidant activity have been reported by Zhao, Xu, Ye and Dong [108]. An extraction time of 75 min at 80 °C, and 100 W US power resulted in the greatest yield (10.9 mg/g). The obtained polysaccharide by US treatment possessed considerable antioxidant activity against DPPH, hydroxyl, and superoxide free radicals [108]. Similarly, the highest yield of polysaccharides from hibiscus leaves was obtained using US power of 93.59 W for 25.71 min at 93.18 °C. Under these conditions, the extracted polysaccharide content was increased by 10% [109]. A study used US (25 kHz, 50–70 W, 50–70 °C, and time range of 10–30 min) for extraction of bioactive polysaccharides from mulberry (*Morus Alba*) leaves proved higher yields and lower water/raw material ratio compared to micro-wave-assisted extraction method [110].

**Fig. 5:**
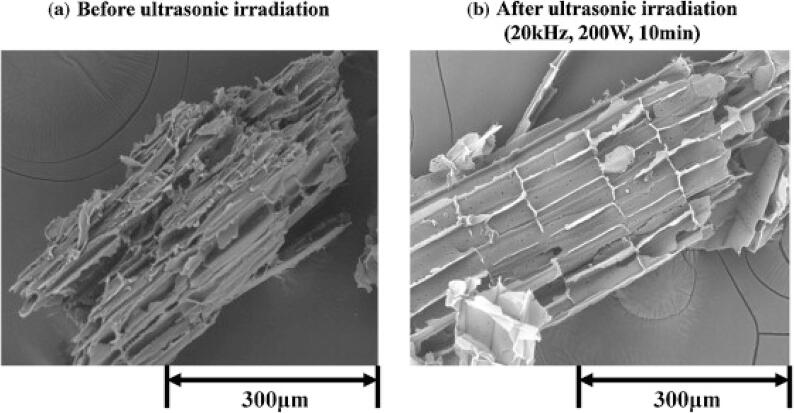
Scanning electron microscope image of Kenaf: (a) Before ultrasonic irradiation (b) After ultrasonic irradiation (20 kHz, 200 W, 10 min) [45] (License Number: 4958670625442).

For cellulose carbohydrate molecule, Nakayama and Imai [45] found that US pretreatment (20 kHz, 200 W, 10 min) can enhance the enzymatic hydrolysis of kenaf herb (*Hibiscus cannabinus*) cellulose to produce glucose sugar. In which, the authors reported that US induced a higher association of cellulase and cellulose by removing the covering materials on the kenaf cellulose, as could be seen in Fig. 5.

Joshi and Gogate [52] reported that US horn (20 kHz, 100 W, and 60 °C) enhanced tea acid hydrolysis for the production of reducing sugars. In which, it reduced the reaction time from 120 to 60 min with a high yield of reducing sugars (24.75 g/L). Also, they reported that US combined with oxidants such as H_2_O_2_ effectively decreased the acid hydrolysis time of the tea polysaccharides through facilitating the breakage of lignin, which increased the rate of reducing sugars production from tea powder [52].

### 4.10 Effect of US on herbal extract activity against pathogenic bacteria

**Fig. 6:**
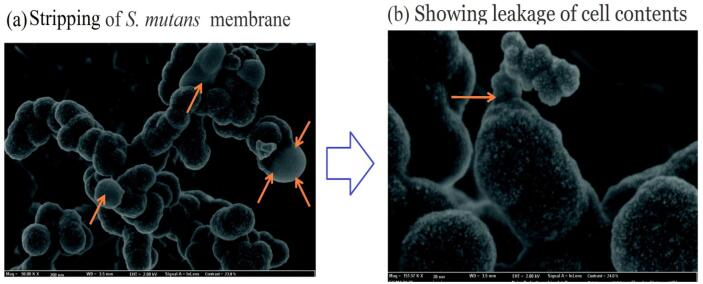
Field Emission Scanning Electron Microscopy images of S. mutans following interaction with US of *Tagetes erecta* flower extracts showing (a) stripping of membrane (b) showing leakage of cell contents (indicated using pointers) [112] (License Number: 4958671204565).

Intense US treatment and long contact times are required to inactivate microorganisms. For instance, to inhibit *Staphylococcus aureus*, a US treatment of 187 min and 150 W, 20 kHz is required [111]. Also, Kazibwe, Kim, Chun and Gopal [112] studied the US (20, 60 kHz; 200, 300 W; 2 min) assisted extraction effects of *Tagetes erecta* herb on their antimicrobial activity. They subjected two different bacterial strains to these extracts (Streptococcus mutans and Pseudomonas aeruginosa). They observed that the extracts by US showed significantly higher inhibition of the two bacteria compared to the hot water extract. Also, they used field emission scanning electron microscope imaging to indicate why the antibacterial increase was happened by US. In which they observed high membrane damage, as observed with Streptococcus mutans (Fig. 6). The bacterial cell sensitivity may be due to the changes in cell surface peptidoglucans adherent. In general, most micro-organisms showed greater sensitivity to US at temperature over 50 °C [113].

Extracted polyphenols from *Erodium glaucophyllum*, which is a Mediterranean herb, exhibited higher antimicrobial activity against *Salmonella enterica*, *Staphylococcus aureus*, and *Listeria* as well as antiviral activities especially against hepatitis A and murine norovirus compared to conventional extraction methods [49].

## 5 Critical analysis of US and other emerging technologies

Compared to other technologies, US technologies show high potential in the field of herbal science due to several unique advantages found in the US. From a financial point of view, it is easier and less expensive to scale-up the US technology compared to other techniques like microwave (MW), pulsed electric fields (PEF), high voltage electric field (HVEF), and high-pressure processing (HPP) methods [32]. Further, the technology generates better yield and thus improves the economics of the extraction process. For instance, Tsaltaki, Katsouli, Kekes, Chanioti and Tzia [42] compared the recovery of bioactive compounds from Damiana leaves (*Turnera diffusa*) by using US, MW, heat reflux (CON), and soxhlet (SOX) extraction methods using 50% ethanol. In that study, US (20 kHz 40 °C, and 15 min) achieved the highest total phenolic yield 203.96 mg GAL/ dry leaves (DL), compared to MW (300 W, 50 °C, and 15 min) and SOX (2.4 × 10^6^ Pa, 100 °C, and 6 h) with 191.36 and 161.62 mg GAL/DL. Similarly, the US increased (*p* < 0.05) the Ginsenside recovery from ginseng (*Panax ginseng*) herb by 31.1%, 19.5%, and 12.1% compared to CONV, MW, and SOX methods, respectively. Additionally, Panadare, Gondaliya and Rathod [106] found that US pre-treatment (150 s at 30 W) increased oil yield by 11% from *Annona squamosal* herb compared to CON methods.

Carbone, Macchioni, Petrella and Cicero [114] compared the use of US and MW for the extraction efficiency of bioactive compounds from hop herb (*Humulus lupulus*). They mentioned that MW (2.45 GHz, 2.4 × 10^6^ Pa, 210 °C, and 1 min) resulted in a higher extraction power for its phenolic contents (95 mg GAL/g) than US (40 kHz, 25 °C, and 30 min) with 25 mg GAL/g. This study suggested that in terms of yield, the efficacy of the US will depend on the plant/ material used, and thus optimization reported in the literature cannot be extrapolated to other materials. In the study of Carbone and colleagues [112], it is important to highlight that US treatment did not use high power like MW (2.45 GHz, 210 °C, 2.4 × 10^6^ Pa), which could have a negative effect on the phytochemicals structure and function. In terms of selectivity, treating rosemary leaves with US (19.5 kHz,140 W) showed remarkably high (*p* < 0.05) recovery of carnosic acid and rosmarinic acid contents (13%, 6.8% of the dried extract) compared to MW (100 °C, 20 bar) [32]. However, MW showed tendency of higher (*p* > 0.1) total terpenoid (28 mg/gDL) compared to US with 27.5 mg/g DL [32].

In a recent study, Nguyen, Gavahian and Tsai [67] compared the effects of US (150 W, 20 min) with conventional (CON), high voltage electric field (HVEF, 4000 kV m^−1^ min), HPP (300 MPa, 3 min) and their combinations treatments on Gac (*Momordica cochinchinensis*) leaves. The authors found that US resulted in the highest (*p* < 0.05) total chlorophyll content (TCC) recovery with 13.67 mg/g DL followed by HPP (12.65 mg/g DL), and then CON (12.58 mg/g DL). Similar results were observed for Stevia (*Stevia rebaudiana*) leaves, in which, the highest TCC was obtained using US and HVEF treatments (20.7 and 20.4 mg/g, respectively) [115].

On the other hand, combined emerging technologies (e.g., US and HPP) are considered superior to individual methods alone. For example, the combination of US and HPP increased (*p* < 0.05) the total flavonoids extraction from Gac leaves to 623 mg QE/100 g DL compared to US and HPP with 592 and 582 mg QE/100 g DL. Furthermore, the inhibitory effect of α-amylase of the combined technique was increased up to 37% compared to US and HPP with 34% and 29% [67]. The better outcome achieved by the combined technologies can be explained by the more efficient disruption of plant cell walls by US and the improved diffusion caused by HPP that enhances the release of bioactive compounds compared to their individual potentials [[83], [84], [116]]. A method combined US with MW confirmed that they could enhance the oil extraction from tea (*Camelia sinensis*) seeds. In that study, an oil yield of 31.52% was obtained under optimum extraction conditions of MW (440 W), US (550 W), at 70 °C for 38 min compared to MW and US methods (27.45% and 25.13%, respectively) [77]. Similarly, Tzima, Brunton, Lyng, Frontuto and Rai [117] found that PEF (5 kW, 1.1 kV cm^−1^, 30 μs), as a pre-treatment step, enhanced (*p* < 0.05) the phenolic extraction and their antioxidant potential of fresh rosemary and thyme herbs by US (200 W, 13 min). The reason could be due to the rearrangement of the phenolic molecules electric ion by PEF and PEF ability to electroporate the cell envelopes, which facilitate the improved recoveries of the US extracted bioactive compounds [117].

## 6 New technologies combined US with herbs and their compounds

### 6.1 Ultrasound with herbal phytochemicals in nanotechnology

The application of US to incorporate herbal phytochemicals in nanotechnology showed promising potential to stabilize bioactives, improve functionality and bioavailability, and delivery. High-intensity US and clove essential oil were used for producing nanofiber hydrogel using cellulose nanofiber. In this study, US (20 kHz, 4 °C) strongly increased the clove essential oil entrapment efficiency by 34%, cell viability rates by 74–101% to human gingival fibroblast cells, water retention, and color characteristics of the prepared hydrogel [40]. *Citrus limon* leaves extract played effectively their role as reducing, capping, and stabilizing agent for forming silver and cadmium oxide Ag:CdO photocatalyst nanotubes and nanospheres [46]. The phytocomponents of *Citrus limon* played an important role against radicals formed by US during nanocomposite formation [46]. Meanwhile, Sathya, Saravanathamizhan and Baskar [47] demonstrated that coriander leaves extract can be used as a reducing agent in iron oxide nanoparticle formation by US assisted technique. The authors reported that the formed nanocomposite had substantial antimicrobial activity against pathogenic bacteria such as *Staphylococcus aureus* and *Micrococcus luteus* that was higher than iron oxide nanoparticle synthesized by the traditional method.

Taha, Modwi, Elamin, Arasheed, Al-Fahad, Albutairi, Arasheed, Alfaify, Anojaidi, Algethami and Bagabas [21] used US for synthesizing zinc oxide (ZnO) nanostructures by using *Hibiscus sabdariffa* extract as a reducing and a stabilizing agent. In that study, the structure morphology, and photocatalytic activity were tested. X-ray diffraction (XRD) confirmed that hibiscus phytochemicals reduced ZnO crystallite size from 40 to 31 nm. Also, as a consequence of band gap reduction and surface area increase, the nanostructures showed better photocatalytic degradation performance with the hibiscus extract. In another study that investigated the use of *Hibiscus tiliaceus* chlorophyll with US to synthesis multi-walled carbon nanotubes [56]. The authors observed an increase in the interaction potential and covalent bonds formation with increasing of US time which led to an increase in the thickness of the nanotube by 160%. Thus, hibiscus phytochemicals are one of the promising approaches in the fabrication of nanoparticles [56].

### 6.2 Ultrasound with acoustic-based sensors and biosensors for the chemical composition of herbs

**Fig. 7:**
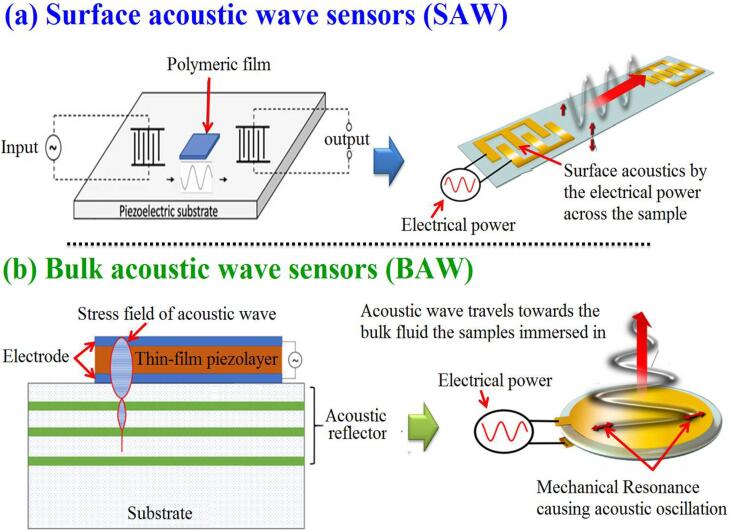
Graphic depicting in general terms the processes for the generation of surface and bulk acoustic waves [[118], [119]] (Copyright permissions: 1093837, 201130–008292).

The use of US for herbal chemical composition measurement and to draw chemical images of plant tissues and visualizing their biomolecules has become one of the hot scientific research areas. An acoustic wave sensor typically consists of a piezoelectric substrate (eg. quartz crystal), coated with sensing material (polymeric film), and two interdigital transducers (one input and one output) are commonly used for chemical composition purposes [118]. There are three different types of these kinds of sensors (Surface acoustic wave sensors (SAW), Bulk acoustic wave sensors (BAW), and Micro/nano-acoustic biosensors) [119]. The acoustic wave propagates on the surface of the substrate is called SAW, while the wave propagates through the substrate is called BAW (Fig. 7) [120]. For example, Sharma, Ghosh, Tudu, Sabhapondit, Baruah, Tamuly, Bhattacharyya and Bandyopadhyay [121] used BAW sensors based on quartz crystal microbalance (QCM) to detect tea aroma (e.g., linalool, geraniol, linalool oxide, Methyl salicylate, and Trans-2-hexenal) during its fermentation process. Zheng, Gao, Zhang, Li, Yu and Hui [122] used SAW as a rapid determination method to study Chinese quince (*Cydonia oblonga Miller*) freshness. They mentioned that using of SAW validated a high predicting accuracy (R^2^ = 0.987). On the other hand, there are still some limitations to these kinds of sensors. For example, QCM sensors have complex circuitry, poor signal-to-noise ratio, and can be influenced by humidity [118].

Micro/nano-Acoustic Biosensors are frequently used to enhance the activity of specific biomolecules such as enzymes for increasing the detection sensitivity. These biosensors are based on a unique class of air-filled protein nanostructures called gas vesicles that vibrate in response to US waves [123]. The principle of using acoustic-based biosensors is based on coupling the measurement nature (like analyte adsorption) as a modulation in the physical properties of the acoustic wave (like US frequency and velocity) that could be correlated with the analyte concentration [119]. Existing molecular biosensors, based on fluorescent emission, have limited utility due to the scattering of light and the interference with their phytochemicals' fluorescents. The use of US can easily image deep tissue with high spatiotemporal resolution. Jiang, Jin and Gui [124] used US-assisted solvothermal reaction for bio-imaging of plant zinc-ion by using quantum dots technology. The authors suggested that the viability of the technique could be used for in-vitro cell imaging and in-vivo imaging of natural plants.

### 6.3 Ultrasound and new chemical assisted extraction of herbal elements and heavy metals

Ultrasound is commonly used in phytochemicals and macromolecules (e.g. protein, and polysaccharide) extractions [25]. However, its beneficial applications in the field of elemental or metal analysis are not well established [125]. A study on heavy metals from herbal medicines such as Hoodia, Shirafza, and Dineh herbs used US-assisted emulsification microextraction (USAEME) to extract Lead, chromium, and cadmium [126]. In that work, the authors confirmed that USAEME is an efficient, rapid, inexpensive, and eco-friendly method for the extraction of macro-elements from herbal medicinal plants [126]. A recent US-assisted method (20 kHz, 80 °C, 50 min) for extraction and determination of trace and ultra-trace impurities (Pb, Cd, Cr, Mn, Fe, Cu, Zn) from 7 plant edible oils including Mustard (*Mustum ardens*) [127]. They mentioned that US-assisted extraction of trace elements efficiency was increased by increasing pH.

**Fig. 8:**
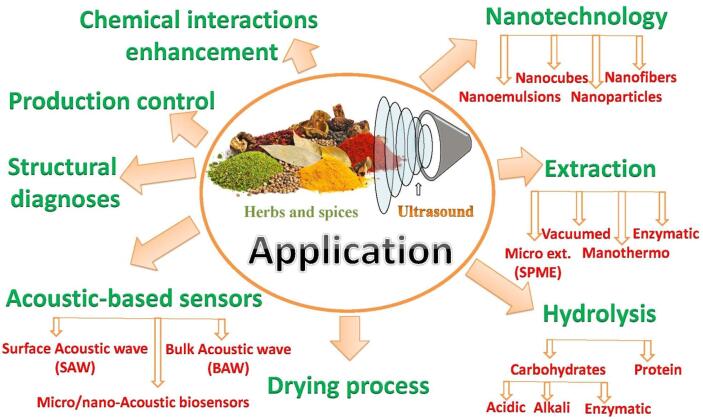
Principle applications between ultrasonic and herbal science and technology.

Fig. 8 summarizes the different common applications that use US in herbal science and technology. In which all these technologies could be improved through the combination of both two fields.

## 7 Conclusion and future directions

Several studies have documented the efficacy of US for the replacement, enhancement, and improvement of various conventional processing techniques in the herbal field. Most of the reports showed that US (25–50 kHz) increases the yields of polyphenols, carotenoids, flavonoids, and essential oils depending based on the used temperature, pressure, and duration parameters. The cavitation's effects on different granules induced by ultrasonication (20–40 kHz, <300 W) further facilitate the chemical, physical, and enzymatic reaction efficiency for herbal bioactive polysaccharide and protein extractions. However, higher power intensity (400–600 W) significantly oxidized and degraded some phytochemicals like (all-E)-astaxanthin carotenoid. The high-frequency US (>100 kHz) is used to obtain chemical information about herbal products. The future perspective will be to combine US and herbal phytochemicals with other technologies like nanosensors and biosensors for forming advanced materials that have unique characters. In which, the optimization of the US parameters for further application development on a large scale level is a very important key role in the herbal industry. Also, US transducers' energy should be further standardizing to present a better green way compared to the commercial methods.

## Declaration of Competing Interest

The authors declare that they have no known competing financial interests or personal relationships that could have appeared to influence the work reported in this paper.
